# Improved Shear Strength Equation for Reinforced Concrete Columns Retrofitted with Hybrid Concrete Jackets

**DOI:** 10.3390/ma16103734

**Published:** 2023-05-15

**Authors:** Kyong Min Ro, Min Sook Kim, Young Hak Lee

**Affiliations:** Department of Architectural Engineering, Kyung Hee University, Deogyeong-Daero 1732, Yongin 17104, Republic of Korea; kyongmin@khu.ac.kr (K.M.R.); kimminsook@khu.ac.kr (M.S.K.)

**Keywords:** seismic retrofitting, concrete jacketing, cyclic loading, shear equation

## Abstract

The adequacy of retrofitting with concrete jacketing is influenced by the bonding between the old section and jacketing section. In this study, five specimens were fabricated, and cyclic loading tests were performed to investigate the integration behavior of the hybrid concrete jacketing method under combined loads. The experimental results showed that the strength of the proposed retrofitting method increased approximately three times compared to the old column, and bonding capacity was also improved. This paper proposed a shear strength equation that considers the slip between the jacketed section and the old section. Moreover, a factor was proposed for considering the reduction in the shear capacity of the stirrup resulting from the slippage between the mortar and stirrup utilized on the jacketing section. The accuracy and validity of the proposed equations were examined through a comparison with the ACI 318-19 design criteria and test results.

## 1. Introduction

Concrete jacketing is an effective seismic retrofit method to improve strength and rigidity by enlarging the cross-section of reinforced concrete columns. The seismic performance of a retrofitted member with a concrete jacket is affected by the reinforcement in the jacketing section, the compressive strength of concrete, and the bonding between the old section and the jacketing section. Therefore, recent studies have been conducted on seismic hooks and steel wire mesh (SWM) to improve the shear performance of the jacketing section and on the use of dowels to constrain the jacketing section and the old section [1,2,3]. Many studies focused on improving the strength of the jacketing section using ultra-high-performance fiber-reinforced concrete (UHPFRC) have been conducted [4,5,6,7]. UHPFRC can show excellent strength, ductility and durability by lowering the water-binder ratio (W/B) by 20% and mixing high-powder admixtures and high-strength steel fibers. The thickness of the jacketing section can be reduced when using such a high-performance material, and the strength and ductility of the retrofitted members were effectively improved. However, UHPFRC, with less water, has a large amount of admixture and no coarse aggregate compared to conventional concrete, which results in high self-shrinkage and a high risk of shrinkage cracking. In a previous study [8], a new hybrid concrete jacketing method was proposed with non-shrinkage mortar in which shrinkage was suppressed by adding an anti-shrinkage admixture. Steel fiber was mixed into the non-shrinkage mortar to enhance the strength of the jacketing section. Conventional concrete jacketing methods use a lot of reinforcement (such as dowels or cross ties) to improve bond capacity, but this reduces workability. Hybrid concrete jacketing methods involve steel wire mesh (SWM) and steel grid reinforcement (SGR). The welded SWM is attached to the surface of the old section to improve the adhesion between the old section and the jacketing section. This makes it possible to omit the process of chipping the concrete surface, thereby improving workability compared to the conventional concrete jacketing method.

**Figure 1:**
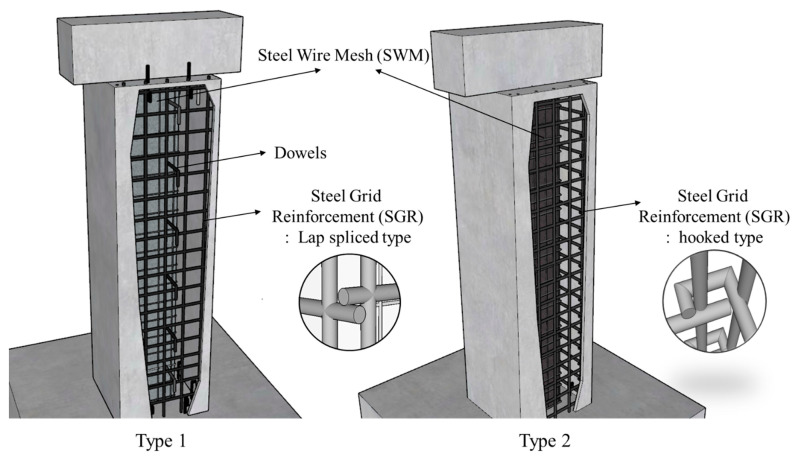
Types of hybrid concrete jacketing methods.

The hybrid concrete jacketing method is divided into two types according to the reinforcement in the jacketing method, as shown in Figure 1. Type 1 is easy to manufacture as welded reinforcing bar grids, but the confinement is reduced, so a small number of dowels are used to improve the bonding capacity between the old section and the jacketing section. Type 2 is a method involving making a hook at the end of the SGR. As compared to seismically designed transverse reinforcement with 135-degree hooks proposed in the ACI 318-19 standard [9], this construction is simpler and less hook loosening occurs, so excellent seismic performance can be expected. When seismic retrofitting a reinforced concrete structure with concrete jacketing, the most important factor is whether a slip occurs at the interface between the old section and jacketing section. Slip occurs at the interface between the core and the jacket concrete if the bonding is not properly secured when a load is applied to the reinforced concrete columns, so the seismic performance of the jacketing section is expected to be unacceptable. There are limited published studies on the contact surfaces of reinforced concrete members and jacketing [6]. Furthermore, when dealing with members having connections, it is important to conduct investigations to identify potential factors that could impact the monolithic behavior, such as slippage [10,11,12,13]. Psycharis and Mouzakis [10] examined the effect of dowel diameter, the number of dowels, and the placement of dowels from the edge of the section on the shear behavior of precast members under different loading patterns. The experimental results indicated that the resistance of the connection under cyclic loading was only half of that under monotonic loading, and the thickness of the cover concrete in the dowel installation direction was found to be related to dowel slippage, which was identified as a factor affecting the shear performance. Therefore, this study experimentally analyzed whether the hybrid concrete jacketing method proposed in a previous study [8] can ensure the appropriate bonding capacity and proposed a shear strength equation for reinforced concrete columns retrofitted with a hybrid concrete jacket.

## 2. Experimental Program

**Figure 2:**
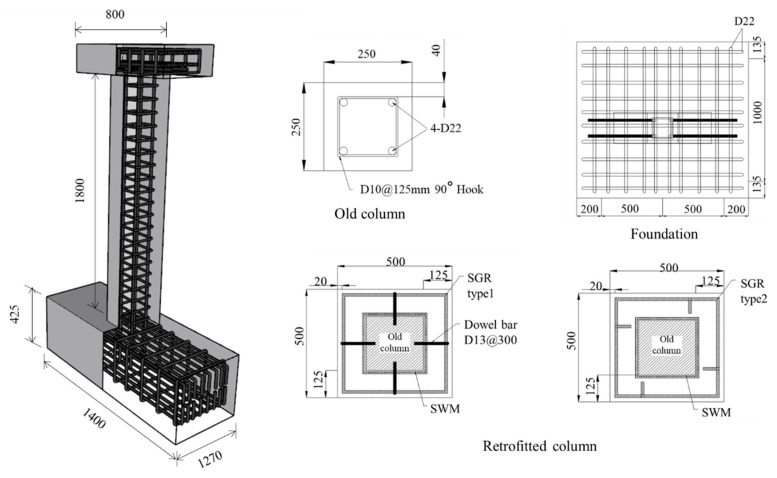
Specimen details (units: mm).

To analyze the seismic performance of the hybrid concrete jacketing method, two test specimens of reinforced concrete columns without seismic design and three specimens retrofitted with hybrid concrete jackets were fabricated and subjected to a cyclic loading test. The details of the specimens are shown in Figure 2 and Table 1. The cross-section of the reinforced concrete column without seismic design was 250 mm × 250 mm, and the height was 1800 mm. For the jacketing section, 4 sides were retrofitted with a thickness of 125 mm by referring to the design method for the concrete jacketing section presented in Pennelis and Kappos [14]. The upper beam is for applying the axial and lateral load, the cross-section was 250 mm × 250 mm, and the length was 800 mm. The specimen was cast on a foundation that was 1400 mm × 1270 mm × 450 mm. The foundation was fastened to a strong floor through high-tensile bolts. The reinforcement was designed in accordance with the ACI 318-19 design standard. Four deformed bars with diameters of 22 mm and 90-degree closed external stirrups with diameters of 10 mm were placed at spacings of 125 mm in the column. The SWM was placed on the jacketing section using steel wire with a diameter of 10 mm, and the SGR, which served as the longitudinal bars and hoops of the column, was manufactured off-site using deformed reinforcing bars with diameters of 13 mm. The compressive strength of the concrete and the non-shrinkage mortar cast on the jacketing section were 24 MPa and 30 MPa, respectively. The yield strength of reinforcing bars placed in the old column and jacketing section was 400 MPa. In the hybrid concrete jacketing method, a non-shrinkage mortar was mixed with steel fibers to improve the structural performance of retrofitted members. The steel fiber was developed for non-shrinkage mortar and had a diameter of 0.34 mm, a length of 18 mm, and a tensile strength of 1250 MPa. Steel fiber content was designed to be 1.5% to ensure excellent performance and workability according to studies of the effect of steel fiber content on structural performance [15,16,17,18]. Table 2 summarizes the material properties used in the manufacture of the specimens. 

**Figure 3:**
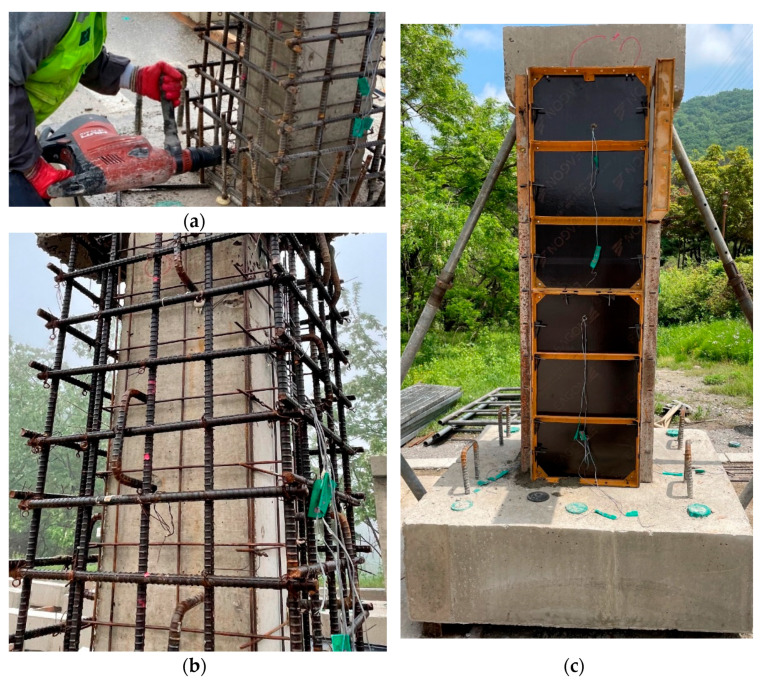
Retrofitting process of a column with the hybrid concrete jacketing method. (**a**) Placing SWM and drilling; (**b**) Installation of reinforcement in jacketing section; (**c**) Formwork of jacketing section.

The manufacturing process of the specimens retrofitted with the hybrid concrete jacketing method is presented in Figure 3. The first step involves wrapping SWM around all four sides of the old column to enhance the bonding capacity between the old column and the jacketing section. The concrete core was fully confined using SWM, and strips of SWM were tightly fastened with steel wires. Subsequently, chemical anchors were installed by drilling the old column to place the SGR or dowel bar. Dowel bars were not used in the case of the specimen with Type 2 detail. Finally, a formwork was constructed on the jacketing section and non-shrinkage mortar mixed with steel fibers was poured. Chipping is a crucial process in conventional concrete jacketing that roughens the section of the old column. However, the hybrid concrete jacketing method improved adhesion performance by bonding SWM to the old column. Therefore, it was possible to omit the chipping process, which generates dust and hinders workability. 

**Figure 4:**
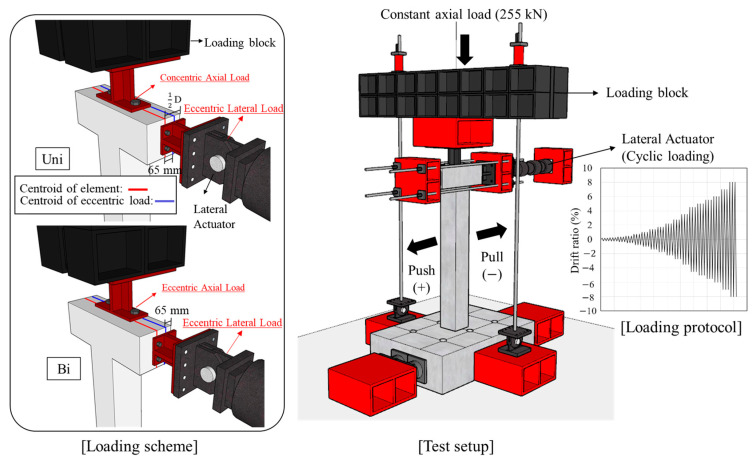
Test setup and loading protocol.

**Figure 5:**
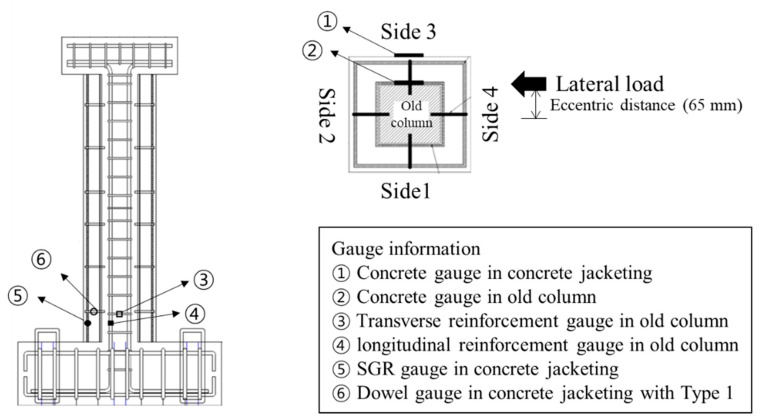
Locations of gauges.

In this study, a cyclic loading test considering axial load, lateral load, and torsional load was conducted to simulate actual seismic load. The test setup and the quasi-static loading protocol are shown in Figure 4. Details related to the loading setup and protocols are provided in a previous study [8]. The cyclic lateral load was applied through the horizontal actuator, and it was increased gradually from a drift ratio of 0.2% until the test was terminated. The definition of drift ratio is the ratio of the lateral displacement to the height from the bottom of the column to the loading point. A constant axial load of 255 kN, which is 17% of the axial load capacity, was applied. An eccentric load was applied to generate torsion with single (unidirectional) or multi-directional (bidirectional) loads. When a compressive force is applied at a location beyond the core of a section, tensile stress is induced in addition to compressive stress, and concrete is especially vulnerable to tensile stress. This study induced the tensile stress on the specimens to simulate extreme conditions during an actual earthquake by applying a load at a location beyond the core of a section. An eccentricity of 65 mm was set, considering the core of a section (1/6 of the section dimension for a rectangular section). To measure the strain of steel reinforcement and concrete, strain gauges were installed near the plastic hinge of the column where the damage is expected to be concentrated, as shown in Figure 5. As shown in Figure 5, the front sides of the specimen were designated Side 1, and the elevations were classified by naming Sides 2, 3, and 4 in the counterclockwise direction to identify the direction.

## 3. Experimental Results

### 3.1. Load-Displacement Relationships

**Figure 6:**
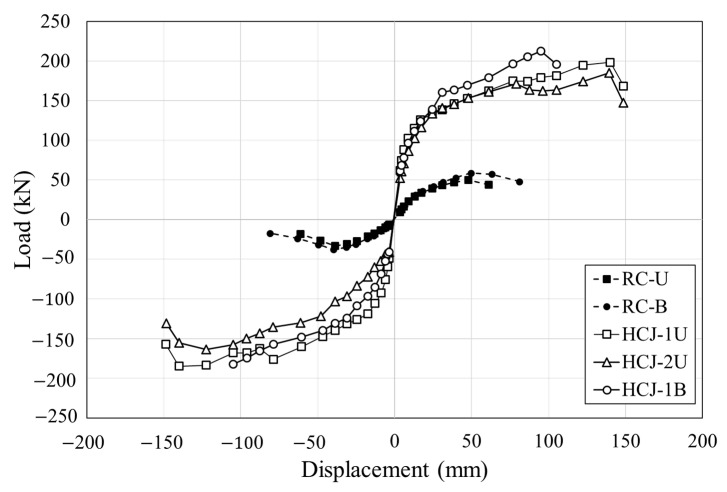
Backbone curve for specimens.

The load-displacement curves for each specimen are shown in Figure 6. Torsion was induced in all specimens, and shear cracks occurred at the bottom of the column, indicating shear failure. To confirm the seismic performance of hybrid concrete jacketing, the load at the occurrence of significant cracking and maximum load are summarized in Table 3. The maximum load of the reinforced concrete column without seismic design and the specimen retrofitted with hybrid concrete jacketing were compared under the same loading scheme. When unidirectional loading was applied, the maximum load of HCJ-1U was 3.9 times that of RC-U, and the maximum load of HCJ-1B was approximately 3.6 times that of RC-B when bidirectional loading was applied. This study confirmed that the hybrid concrete jacketing method is effective for seismic retrofit regardless of the loading scheme. When unidirectional loading was applied, the torsion induced in the specimen increased compared to bidirectional loading because the axes of axial force and lateral force did not coincide. If torsion is induced in reinforced concrete columns, shear cracking and concrete spalling are generally observed at the bottom of the column, and brittle failure occurs under extreme torsion. The hybrid concrete jacketing method can increase the strength and rigidity by enlarging the cross-section of the column, effectively resisting torsion because the steel fiber suppresses the propagation of shear cracks. Therefore, the difference in maximum load under unidirectional loading and bidirectional loading was insignificant, and there were no significant shear cracks or brittle failures.

**Figure 7:**
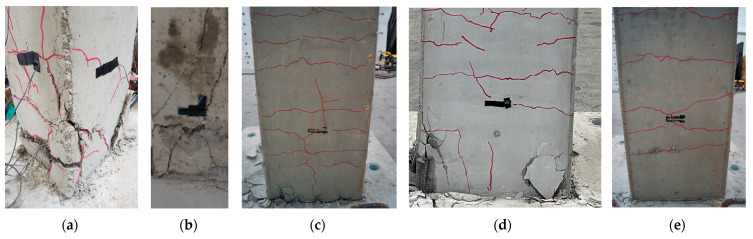
Failure of specimens at the bottom of the column: (**a**) RC-U, (**b**) RC-B, (**c**) HCJ-1U, (**d**) HCJ-2U, and (**e**) HCJ-1B.

HCJ-1U and HCJ-1B retrofitted with Type 1 reached the maximum loads at 8.5% and 6% of the drift ratio, respectively, and the maximum loads were 198.32 kN and 202.45 kN, respectively. The maximum load of HCJ-2U retrofitted with Type 2 was 7% lower than that of HCJ-1U retrofitted with Type 2 under the same load scheme. Unlike Type 1, in which dowels were placed to improve the bonding performance between the old section and the jacketing section, Type 2 omitted additional reinforcement by placing SGR with hooked details in the jacketing section. The bonding capacity between the old section and the jacketing section was reduced in Type 2 compared to Type 1, resulting in relatively more slip. However, since similar seismic performances were observed compared to the simple construction process, this study confirmed that if Type 2 details are used, seismic performance can be secured without using additional shear reinforcement such as dowels. These results can be observed through the failure patterns of each specimen shown in Figure 7.

### 3.2. Torsional Moment versus Twist Response

**Figure 8:**
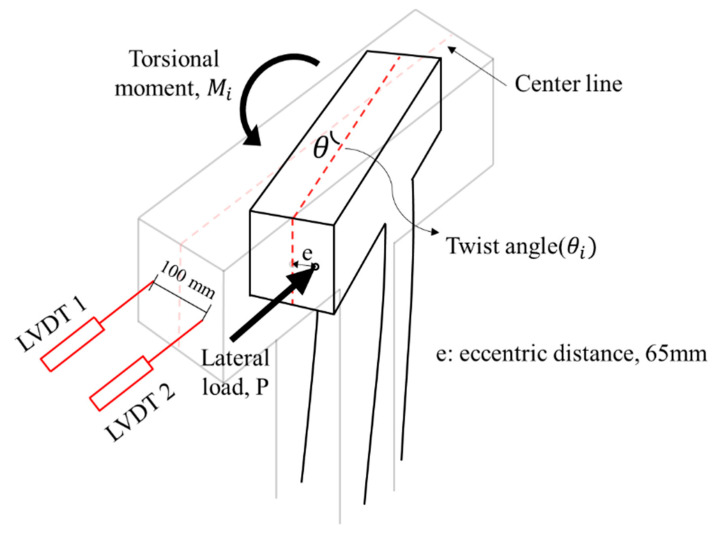
Torsional moment and twist.

The torsional moment and twist were obtained from LVDTs and strain gauges, as shown in Figure 8. These were calculated as shown in Equations (1) and (2).

(1) $$M_{i}=P\times \ell \times {\cos\theta }_{i}$$

(2) $$\theta_{i}=\tan^{-1}\left(\frac{\Delta_{2}-\Delta_{1}}{d}\right)$$

Here, $M_{i}$ is the torsional moment generated to the specimen at the i-th drift ratio, P is the maximum load at the i-th drift ratio, ℓ is the eccentric distance, $\theta_{i}$ the twist of the column cross-section, and d is the distance between LVDTs. 

**Figure 9:**
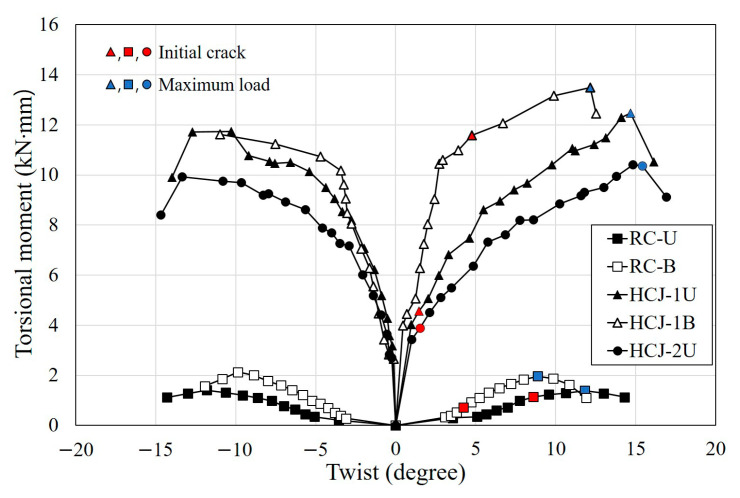
Torsional moment-twist relationships in the specimens.

Torsional moment-twist envelopes are shown in Figure 9. The torsional moment tended to increase gradually until shear cracks occurred in all specimens. The torsional moment decreased dramatically as the shear crack extended and failed. In the case of non-retrofitted specimens, the maximum torsional moment of RC-U under unidirectional loading was 2.13 kN·m, and the maximum torsional moment of RC-B under bidirectional loading was 1.41 kN·m. This result indicated that a degradation in seismic performance of about 60% was observed when a larger torsion was applied. This is a general tendency observed in reinforced concrete columns [19]. Comparing retrofitting with hybrid concrete jacketing and non-retrofitted specimens, the seismic performance improved by 7.8 times when unidirectional loading was applied and 5.8 times when bidirectional loading was applied. HCJ-2U with Type 2 reinforcement was about 80% of the maximum torsional moment of HCJ-2B with Type 1, and the twist increased. This means that the details of the reinforcement in the jacketing section can affect the contact surfaces between the old section and the jacketing section.

### 3.3. Strain

**Figure 10:**
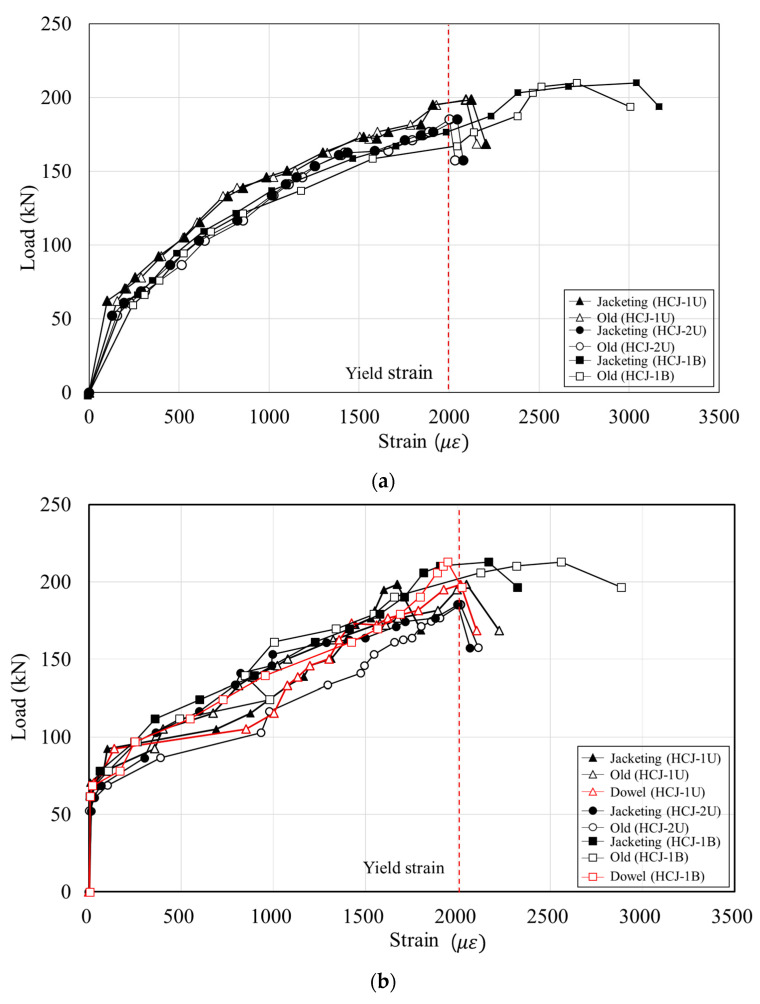
Strain of reinforcement: (**a**) longitudinal reinforcement and (**b**) stirrup.

Strains of the reinforcing bars in the old column and the jacketing section were compared to analyze the integration behavior of the hybrid concrete jacketing method. Figure 10 shows the load-strain relationship of the reinforcing bars of the retrofitted specimens. In HCJ-1U and HCJ-1B, the strain of the longitudinal bars of the old column and the vertical steel bars of the SGR (which act like longitudinal bars) showed a similar tendency until the maximum load. The dowels (which transmit stress to the SGR) also yielded increasing strain until the maximum load, confirming that the SGR and dowels placed in the jacketing section effectively resisted the load regardless of the loading scheme. In HCJ-1U and HCJ-2U, where the reinforcement details varied, the strain distribution of longitudinal reinforcing bars in the old column and the SGR was similar until yielding, and yielding occurred at the point of maximum load. The strain of transverse reinforcement of SGR in HCJ-1U increased gradually as the load increased, and it yielded at the maximum load. However, the strain of dowels increased non-linearly, and it did not yield until the maximum load. This indicated that dowels in Type 1 effectively transferred shear stress to the SGR. The strain of the transverse reinforcing bars of SGR in HCJ-2U also showed a gradual increase and then yielded at the point of maximum load. This means that the hooked end detail of SGR in Type 2 acted as a constraint and helped prevent buckling and shear crack control. Test results showed that columns retrofitted with Type 1 and Type 2 behaved similarly to the monolithic reinforced concrete column under the combined load considering torsion. 

**Figure 11:**
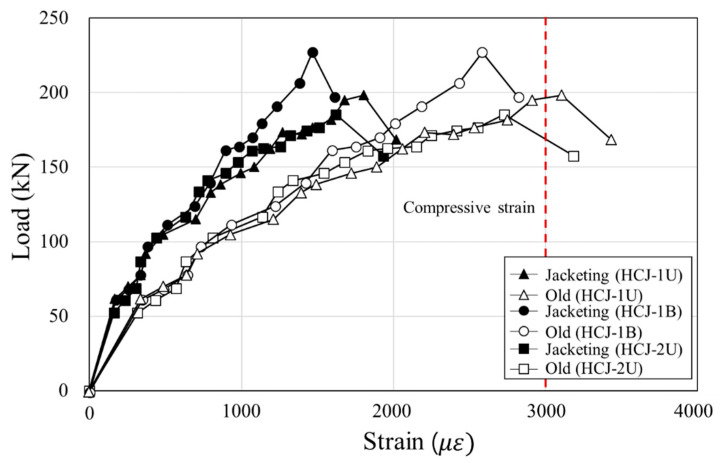
Strain of concrete.

When a reinforced concrete column retrofitted with concrete jacketing behaves like a monolithic column, the difference in concrete strain between the old section and the jacketing section tends to increase proportionally until the ultimate strain [6,20]. However, when slip occurs at the surface between the old section and the jacketing section, the concrete jacket does not resist deformation, and concrete strain at the jacketing section does not increase. Therefore, the bonding capacity of the hybrid concrete jacket was evaluated in this study by analyzing the concrete strain of the surface of the old column and the jacketing section. Figure 11 shows the load-strain relationship of concrete. HCJ-1U and HCJ-2U subjected to unidirectional loading exceeded the ultimate strain of the concrete (0.003) when reaching the maximum load. However, the strain of the concrete in HCJ-1B did not reach the ultimate strain until the experiment terminated. This is because the torsion induced in the specimen was small, and the damage to the column until the failure was not significant. As the drift ratio increased in all retrofitted specimens, the difference in concrete strain between the old section and the jacketing section gradually increased. Compared to the increase in concrete strain in the old section, the increase in concrete strain in the jacketing section was lower. This means that a slip occurred at the interface.

## 4. Proposed Shear Strength Equation

**Figure 12:**
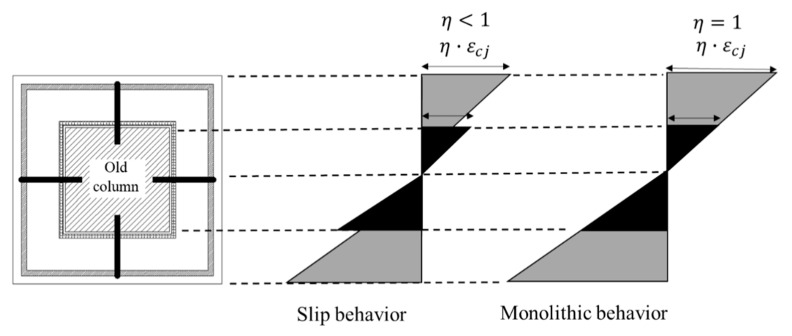
Strain profile.

The seismic performance of a concrete jacketed column depends on the bonding of the old concrete and added concrete. If appropriate shear reinforcement is designed in the jacketed section, a column with a concrete jacket should act monolithically under seismic load. Therefore, the shear strength and behavior of a reinforced concrete column retrofitted with concrete jacketing are predicted, considering the jacketed section to be an equivalent monolithic section [21]. The experimental results confirmed that the SGR of the hybrid concrete jacketing method performed the role of longitudinal reinforcements and hoops. Therefore, the reinforced concrete column retrofitted with hybrid concrete jacketing was an adequately designed shear reinforcement. In the past few decades, the bond-slip model was mainly considered for reinforced concrete strengthened with fiber-reinforced polymer (FRP) to avoid debonding failure. However, recent studies have confirmed that reinforced concrete columns retrofitted with concrete jackets should consider the slip when it takes place along the interfaces between the old section and the jacketing section [20,22,23]. The mechanics of reinforced concrete members retrofitted with a concrete jacket are quite complex. In particular, it is difficult to consider the behavior of the interface between the existing member and the jacket [23]. In this study, the slip coefficient was experimentally determined as a measure of the bonding capacity between the two sections to assess the behavior of the interface between the concrete jacket and the existing members. It is common to measure the slip coefficient experimentally considering various factors such as the strain, material of the jacket and existing member, friction coefficient, and angle. Figure 12 shows the strain profile of the jacketed cross-section. As shown in Figure 12, the slip coefficient, η, is a value that measures the frictional force between the jacketing section and the existing member. It is an indicator of the strength of the bonding capacity between the two sections. The slip coefficient is one of the most important factors in ensuring safe attachment between the jacket and the existing member in the concrete jacketing method. If the old column is considered to be fully confined due to the confinement provided by the jacket, there is no sliding at the interface between the column and jacket. Therefore, the slip coefficient equals 1.0. On the other hand, in a partially confined column, the slip coefficient is less than 1.0, and the concrete strain of the jacketed section decreases by the ratio of the slip coefficient. This value is usually in the range of 0.8 to 1.0, with a higher value indicating a stronger friction force between the jacket and the existing member.

**Figure 13:**
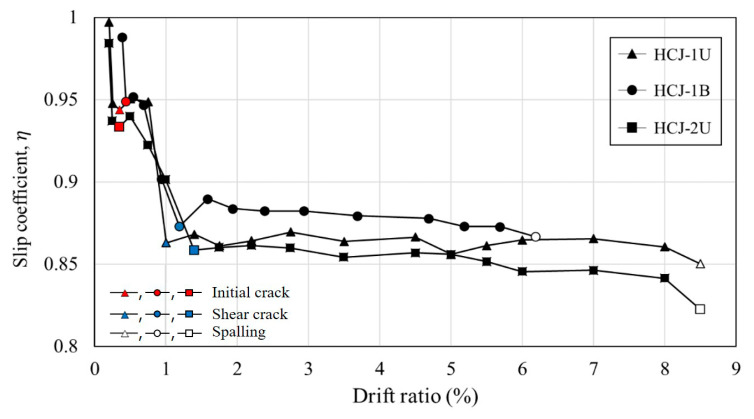
Slip coefficient from concrete strain.

To obtain the slip coefficient, gauges were used to measure the concrete strain in both the old section and the jacketing section, as shown in Figure 5. The gauges were placed at the same location in the cross-section of the existing member and the jacketing concrete, as illustrated in Figure 5. The slip coefficient was calculated based on the difference in concrete strain between the two sections, with a higher value indicating a smaller difference in strain and stronger bonding between the old section and the jacketed concrete section. Figure 13 depicts the slip coefficients obtained by calculating the difference in strain between the old section and the jacketing section for each drift ratio. At the onset of the experiment, the slip coefficient is approximately 1.0. However, all specimens exhibited a trend of decreasing slip coefficient as the drift ratio increased. This is because the torsion acting on the specimen increased according to the drift ratio. In addition, the slip coefficient decreased rapidly from the initial crack occurrence to the point of shear crack occurrence, and the slip coefficient converged after shear cracking. Bonding between the jacketing section and the old section decreased because torsion causes shear cracks in columns. The slip coefficient did not decrease beyond a certain level since the SWM attached to the old section secured the bonding capacity with the jacketing section. In this paper, the smallest value among the slip coefficients measured in the experiment was considered when calculating shear strength to ensure a conservative design. The slip coefficients for the HCJ-1U, HCJ-1B, and HCJ-2U specimens were verified to be 0.86, 0.88, and 0.82, respectively. Due to the misaligned axes of axial and lateral forces during unidirectional loading, HCJ-1U exhibited a smaller slip coefficient than HCJ-1B because the torsional forces were greater. Nevertheless, the discrepancy in slip coefficient between loading schemes was insignificant at approximately 2%. Since it is not feasible to anticipate the loading scheme under actual seismic loads, a conservative slip coefficient of 0.86 was assigned to Type 1. It should be noted that Type 2 had no dowel (unlike Type 1), and it ensured bonding capacity through the hooked details in SGR. As a result, the slip coefficient of Type 2 decreased compared to that of Type 1, with a value of approximately 95% of Type 1.

**Figure 14:**
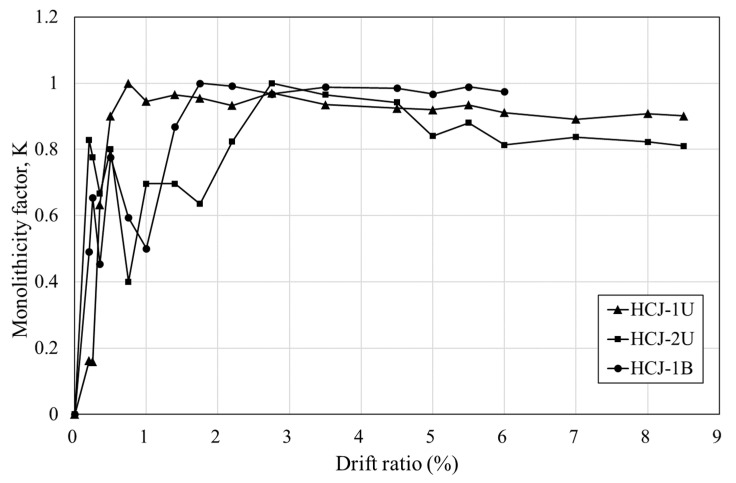
The monolithicity factor from stirrup strain.

This study proposed a novel concrete jacketing method that employs non-shrinkage mortar for concrete jacketing, with the objective of enhancing workability and alleviating the problem of high self-shrinkage that is typically associated with Ultra-High-Performance Fiber-Reinforced Concrete (UHPFRC). It is important to note that the slip of stirrups in concrete can have a significant impact on the strength and durability of columns, particularly in columns that use mortar. This is due to the relatively smooth surface of mortar, which can lead to increased stirrup slippage compared to concrete columns. As depicted in Figure 10, the strain patterns of the stirrups in the old and jacketing sections were similar. Nonetheless, slippage between the mortar and stirrup could potentially occur in the jacketing section, thereby compromising the shear performance of the stirrup. Consequently, the disparity in strains between the old and jacketing sections of the stirrup was analyzed, and this factor was accounted for in the evaluation of the shear strength of the stirrup in the jacketing section. The monolithicity factor (K) was introduced as a measure of bonding performance in the concrete jacketing method to investigate the reduction in shear capacity resulting from stirrup slippage within the jacketing section. The K factor is defined as the ratio of the response index of composite members to the response index of monolithic members with an identical geometry [23]. In this study, a comparison between a monolithic concrete column and a jacketed concrete column having identical geometries was not carried out. Nevertheless, the stirrup of the existing concrete column and that of the concrete jacket were designed to exhibit equivalent shear performance. Hence, the monolithicity factor was determined by computing the ratio of the stirrup strain in the jacketing section to that in the old section. The monolithicity factor was subsequently normalized to a maximum value of 1, and the results are presented in Figure 14. 

During the initial stage of the experiment, the load was carried by the stirrup in the old column until yielding occurred, after which the stirrup within the jackets began to carry the load with an increase in load. Consequently, the monolithicity factor K, which denotes the ratio of stirrup strain in the jacketing section to that in the old section, gradually increased and approached unity in the early stages of the experiment. However, the load carried by the stirrup within the jacketing section increased as the torsional load increased, causing a reduction in friction between the stirrup and mortar in the jacket, resulting in a decrease in K. This phenomenon was observed consistently in all specimens, and K approached a constant value at the maximum load. The loading scheme was closely related to K, and the values of K at the failure for HCJ-1U, HCJ-2U, and HCJ-1B were 0.9, 0.82, and 0.97, respectively. Unidirectional loading led to an increase in torsional load, a reduction in bonding capacity between the stirrup and mortar within the jacketing section, and an increase in shear stress in the jacketing section. Moreover, Type 2 showed a greater decrease in bonding capacity between the stirrup and mortar within the jacketing section than Type 1 because no dowel was used to connect the old and jacketing sections, resulting in more sliding between the two sections and an increase in shear strength within the jacket.

In this study, the shear strength of concrete and stirrup was estimated using the ACI 318-19 design criteria as shown in Equations (3)–(5).

(3) $$V_{n}=V_{c}+V_{s}$$

(4) $$V_{c}=\frac{1}{6}(1+\frac{N_{u}}{14A_{g}})\lambda \sqrt{{f^{\prime }}_{c}}b_{w}d$$

(5) $$V_{s}=\frac{A_{v}f_{yt}d}{s}$$

Here, $A_{g}$ is the gross area of the concrete section, $A_{v}$ and s are the area and spacing of shear reinforcement, $b_{w}$ is the web width of the cross-section, *d* is the distance from extreme compression fiber to the centroid of longitudinal reinforcement, ${f^{\prime }}_{c}$ is the compressive strength of the concrete, $f_{yt}$ is the yield strength of the stirrup, $N_{u}$ is the factored axial force acting on the cross-section of the column, and *λ* represents the influence of lightweight concrete. 

The shear strength equation of the reinforced concrete column retrofitted with hybrid concrete jacketing was proposed as shown in Equations (6)–(8). If slippage occurs between the old and jacketing sections, both sections may not fully exhibit their shear performance under seismic loads. To mitigate this issue, this study proposed a slip coefficient for each type of hybrid concrete jacketing method that considers the level of slippage between the old and jacketing sections based on experimental results. As a result, the contribution of concrete was estimated by multiplying the sum of the shear contributions of the old and jacketed sections by the slip coefficient to evaluate the shear strength of a reinforced concrete column retrofitted with hybrid concrete jacketing. The hybrid concrete jacketing method used mortar instead of concrete in the jacketing section, which can result in increased slip between the reinforcement steel and mortar due to the absence of coarse aggregate. Therefore, the reduction in stirrup shear capacity due to a slip between the mortar and the stirrup should be considered. The shear strength of the stirrup in the jacketing section was determined by multiplying the shear strength of the stirrup in the existing reinforced concrete column by a monolithicity factor, which is a reduction factor. The variable n in Equation (8) indicates the number of pertinent entities drawn from the subsequent options:

(1) When a unidirectional load is applied.
(2) When no additional steel, such as dowels, is provided to enhance the bonding performance between the old section and the jacketing section.

(6) $$V_{n,proposed}=\eta \;\left(V_{c,old}+V_{c,jacket}\right)+V_{s,old}+K\cdot V_{s,jacket}$$

(7) $$\eta =0.86\;\left(\mathrm{for}\;\mathrm{Type}\;1\right),\;0.82\;\left(\mathrm{for}\;\mathrm{Type}\;2\right)$$

(8) $$K=0.9^{n}$$

The experimentally obtained shear strengths were compared with those predicted by the ACI 318 design code and the proposed equation presented in Table 4. The shear strength evaluated based on the ACI 318-19 and the proposed equations neglect the loading scheme applied to the reinforced concrete column. Huang et al. [19] conducted cyclic loading tests on nine reinforced concrete columns with variable load patterns and found that the shear strength of the columns decreased by approximately 60% when subjected to an eccentric lateral force as compared to the case where no eccentricity was present. Accordingly, this study compared the experimental results and the shear strength determined by the design criteria and proposed equations, with the latter being reduced to 60% of their calculated values. The ACI 318 design code, which does not consider slip, was found to overestimate the experimental results by approximately 23%. This was attributed to excessive deformation caused by seismic loads acting on the column, which led to a slip between the old and jacketing sections of the column. However, the proposed equation calculated the shear strength of reinforced concrete columns retrofitted with concrete jackets in detail by considering slip as a factor. The slip coefficient and monolithicity factor were derived by measuring the strain of the concrete and stirrups in the old and jacketing sections and considering the strength reduction due to slip. The proposed equation yielded a shear strength ratio of approximately 1.1 to that obtained from the experiment for all specimens, indicating a conservative prediction. Therefore, the proposed equation was deemed capable of accurately predicting the shear strength of reinforced concrete columns retrofitted with concrete jackets, mitigating the issue of overestimation observed in the existing design code.

## 5. Conclusions

This study investigated the bonding capacity of a reinforced concrete column retrofitted with hybrid concrete jacketing under combined loading. Five specimens were fabricated, and cyclic loading tests were conducted. Based on the test results, the authors proposed a shear strength equation that considers the slip between the jacketed section and the old section. The following conclusions have been drawn:

(1) The hybrid concrete jacketing used non-shrinkage mortar and steel fiber to suppress shrinkage and enhance strength. This retrofitting method improved bond capacity with steel wire mesh and steel grid reinforcement, and this method is divided into two types, Type 1 and Type 2, according to the reinforcement details in the jacketing section. Additional dowels which can improve the bonding capacity were used only in Type 1;
(2) The cyclic loading test results demonstrated that the torsional moment of the jacketed column with Type 2 was approximately 80% of the jacketed column with Type 1. Additionally, the difference in strain between the old section and the jacketed section increased gradually, indicating that a slip occurred between the two sections. Therefore, when applying hybrid concrete jacketing for retrofitting old columns, it should be considered the slippage between two sections and shear resistance capacity according to the types of hybrid concrete jacketing methods used;
(3) The slip coefficient and the monolithicity factor were proposed in this study for reinforced concrete columns retrofitted with hybrid concrete jacketing. The slip coefficient accounts for the bonding capacity between the old and jacketed sections; it was dependent on the type of hybrid concrete jacketing, with Type 1 exhibiting a value of 0.86, while Type 2 had a coefficient of 0.82. The monolithicity factor was proposed as a parameter that accounts for the reduction of the stirrup shear strength resulting from the slip between the mortar and the stirrup in the jacketing section. This factor varies based on the presence of dowel bars and the loading pattern;
(4) In this study, the shear strength equation for reinforced concrete columns retrofitted with the hybrid concrete jacketing was proposed by introducing the slip coefficient and monolithicity factor. In contrast to the ACI 318-19, which overestimates test results by approximately 23%, the proposed equation yielded conservative estimates, underestimating the results by approximately 3%. It was indicated that the proposed equation is more reliable and accurate for evaluating the shear strength of jacketed columns. Overall, the results of this study provided important insights into the use of the hybrid concrete jacketing method for retrofitting reinforced concrete columns, and the proposed shear strength equation could be useful for assessing the structural performance of jacketed columns.

## Figures and Tables

**Table 1:** Details of specimens.

Specimen	Retrofit Method	Loading Scheme	Cross Section [mm]
RC-U	–	Unidirectional	250 × 250
RC-B		Bidirectional	
HCJ-1U	Type 1	Unidirectional	500 × 500
HCJ-2U	Type 2	Unidirectional	
HCJ-1B	Type 1	Bidirectional	

**Table 2:** Mechanical properties of specimens.

	Concrete	Non-Shrinkage Mortar	SFNM	Reinforcement Steel
Compressive strength (MPa)	24	30	40.1	-
Yield strength (MPa)	-	-	-	400
Material	Diameter [mm]	Length [mm]	Aspect Ratio	Tensile Strength [MPa]
Steel fiber	0.34	18	0.019	1250

**Table 3:** Test results.

Specimen	Initial Crack		Shear Crack		Maximum	
	Load (kN)	Drift Ratio (%)	Load (kN)	Drift Ratio (%)	Load (kN)	Drift Ratio (%)
RC-U	15.21	1	43.53	2.2	50.61	3.5
RC-B	13.15	0.75	47.35	1.4	59.48	4.5
HCJ-1U	62.05	0.2	125.96	1	198.32	8.5
HCJ-2U	60.76	0.2	116.54	1	185.23	8.5
HCJ-1B	61.40	0.2	111.47	0.75	202.45	6

**Table 4:** Validity of the proposed equation.

Specimen	$V_{test}\;[\mathrm{kN}]$	$V_{n,ACI}\;[\mathrm{kN}]$	$V_{n,proposed}\;[\mathrm{kN}]$	$V_{test}$ $/V_{n,ACI}$	$V_{test}$ $/V_{n,Proposed}$
HCJ-1U	198.32	210.45	191.43	0.94	1.04
HCJ-2U	185.23		181.53	0.88	1.02
HCJ-1B	202.45		197.70	0.96	1.02

## Data Availability

Not applicable.
